# Supplementary material for: Translational control plays an important role in the adaptive heat-shock response of Streptomyces coelicolor
Source: Nucleic Acids Res. 2018 May 9;46(11):5692–703. doi: 10.1093/nar/gky335 (PMC6009599; doi:10.1093/nar/gky335)
Supplement: Supplementary Data [file gky335_supplemental_files.zip › Bucca_Supplementary_Figure_1.pptx]

## Slide 1
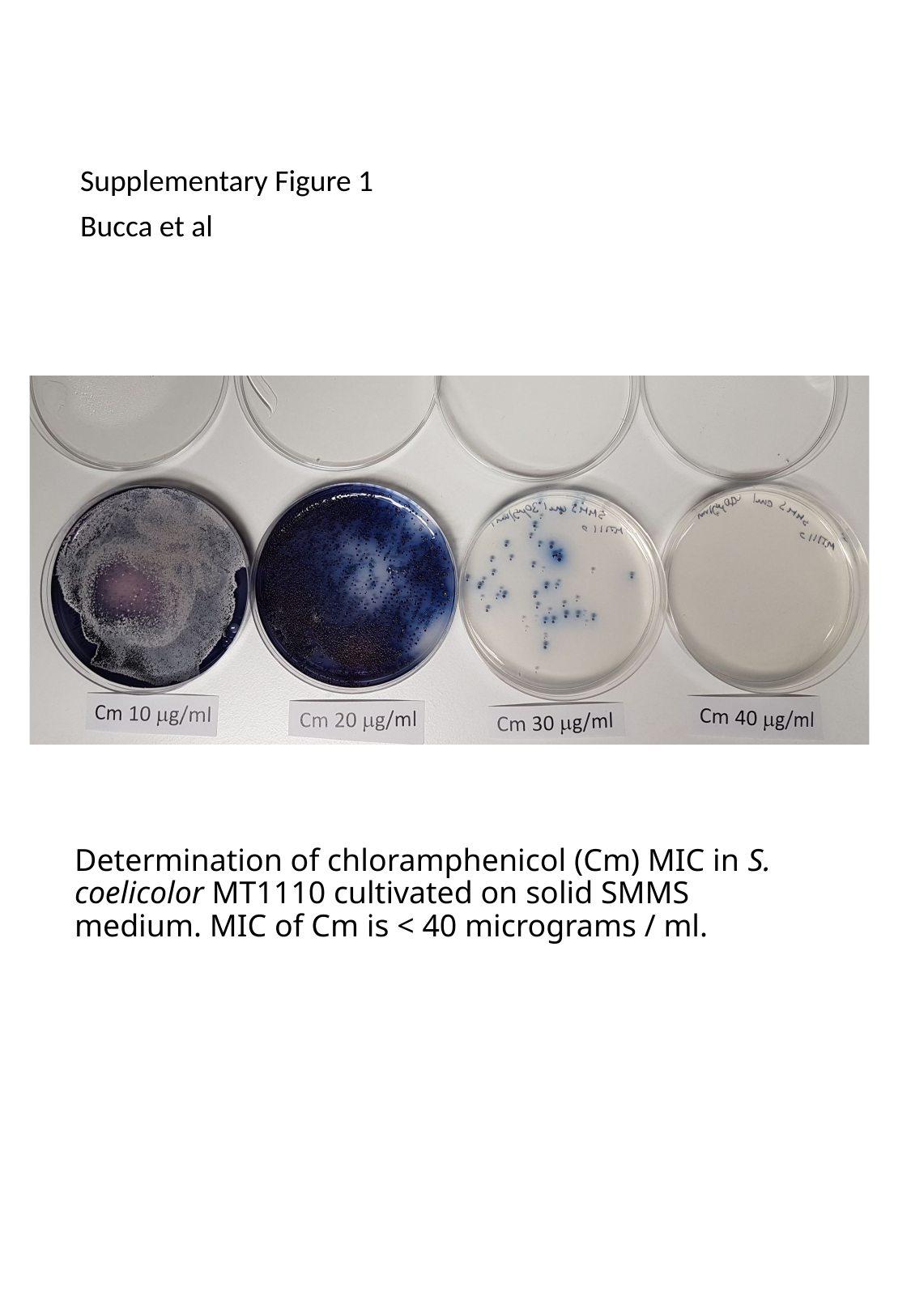

Supplementary Figure 1
Bucca et al
# Determination of chloramphenicol (Cm) MIC in S. coelicolor MT1110 cultivated on solid SMMS medium. MIC of Cm is < 40 micrograms / ml.
