# Supplementary material for: Translational control plays an important role in the adaptive heat-shock response of Streptomyces coelicolor
Source: Nucleic Acids Res. 2018 May 9;46(11):5692–703. doi: 10.1093/nar/gky335 (PMC6009599; doi:10.1093/nar/gky335)
Supplement: Supplementary Data [file gky335_supplemental_files.zip › Bucca_Table S3.docx]

Table S3

|  | Codon | % in TE UP genes (n=73) | % ALL genes  (n=7690) |
| --- | --- | --- | --- |
| Start codon | ATG | 65.7 | 61.7 |
|  | GTG | 30.1 | 35.1 |
|  | TTG | 4.1 | 0.3 |
| Stop codon | TGA | 76.7 | 78.1 |
|  | TAA | 8.2 | 4.4 |
|  | TAG | 15.0 | 17.4 |
