# Supplementary material for: Translational control plays an important role in the adaptive heat-shock response of Streptomyces coelicolor
Source: Nucleic Acids Res. 2018 May 9;46(11):5692–703. doi: 10.1093/nar/gky335 (PMC6009599; doi:10.1093/nar/gky335)
Supplement: Supplementary Data [file gky335_supplemental_files.zip › Supplementary data captions_revised.docx]

**Supplementary data captions**

**Supplementary Data File 1.** (format: .xlsx)

Normalised microarray data and Rank Products analysis of the transcriptome and translatome data reported in this study. The first worksheet, README, details the contents of the data file

**Table S1** (format: xlsx)

Significant translationally enhanced transcripts at 42°C >30°C (74 genes) and 30°C >42°C (3 genes). List of genes (column A); abundance fold changes at 42°C/30°C (74 genes) and 30°C/42°C (3 genes) (column B), probability of false prediction from rank product analysis (column C), significantly different also at transcription at 42°C>30°C where 0 denotes not significant, 1 significantly upregulated and -1 significantly downregulated, protein identifier (column F) protein name (column G) gene name (column H) gene length (column I) gene ontology (columns L-N) and gene name (column R)

**Table S2** (format: xlsx)

Gene lists represented in the Venn Diagram (Fig 3D)

Legend

Gene = SCO number of gene

Product = predicted protein product

Symbol = gene name symbol

Protein.Length = length in amino acid residues of the predicted protein

Significance_Monosome = significance call from rank products analysis of transcript abundance changes in the Monosome fraction following heat-shock: 0=not significant, 1=significantly increased at 42^o^C, -1 = significantly decreased at 42^o^C

Significance_Polysome = significance call from rank products analysis of transcript abundance changes in the Polysome fraction following heat-shock: 0=not significant, 1=significantly increased at 42^o^C, -1 = significantly decreased at 42^o^C

Significance_T = significance call from rank products analysis of transcript abundance changes in the transcriptome following heat-shock: 0=not significant, 1=significantly increased at 42^o^C, -1 = significantly decreased at 42^o^C

Significance_TE = significance call from rank products analysis of changes in TE following heat-shock: 0 = not significant, 1= significantly increased at 42^o^C, -1 = significantly decreased at 42^o^C

Significance_Monosome v T = significance call from rank products analysis of changes in ratio of Monosome:T following heat-shock: 0=not significant, 1=significantly increased at 42^o^C, -1 = significantly decreased at 42^o^C

Significance_Monosome v Polysome = significance call from rank products analysis of changes in ratio of Monosome:Polysome following heat-shock: 0=not significant, 1=significantly increased at 42^o^C, -1 = significantly decreased at 42^o^C.

Table S3 (format: docx)

Start codon and stop codon preferences in the 73 TE genes (note that one of the 74 original TE UP genes is a pseudogene)
